# Management of constipation in patients with Parkinson’s disease

**DOI:** 10.1038/s41531-018-0042-8

**Published:** 2018-03-16

**Authors:** Anna J. Pedrosa Carrasco, Lars Timmermann, David J. Pedrosa

**Affiliations:** 1 Sir Michael Sobell House, Oxford University Hospitals, Oxford, UK; 2 King’s College London, Cicely Saunders Institute, Department of Palliative Care, Policy and Rehabilitation, London, UK; 3 Department of Internal Medicine V, University Hospital of Gießen and Marburg, Gießen, Germany; 4 Department of Neurology, University Hospital of Gießen and Marburg, Marburg, Germany; 5 Department of Psychiatry, University Hospital of Gießen and Marburg, Marburg, Germany

## Abstract

A considerable body of research has recently emerged around nonmotor symptoms in Parkinson’s disease (PD) and their substantial impact on patients’ well-being. A prominent example is constipation which occurs in up to two thirds of all PD-patients thereby effecting psychological and social distress and consequently reducing quality of life. Despite the significant clinical relevance of constipation, unfortunately little knowledge exists on effective treatments. Therefore this systematic review aims at providing a synopsis on clinical effects and safety of available treatment options for constipation in PD. For this purpose, three electronic databases (MEDLINE, EMBASE, PsycINFO) were searched for experimental and quasi-experimental studies investigating the efficacy/effectiveness of interventions in the management of PD-associated constipation. Besides, adverse events were analyzed as secondary outcome. In total, 18 publications were identified involving 15 different interventions, of which none can be attributed sufficient evidence to derive strong recommendations. Nevertheless, some evidence indicates that dietetic interventions with probiotics and prebiotics may reduce symptom burden while providing a very favorable side-effects profile. Furthermore, the use of lubiprostone, macrogol and in the specific case of isolated or prominent outlet obstruction constipation injections of botulinum neurotoxin A into the puborectal muscles may as well be moderately supported. In summary, too little attention has been paid to treatment options for constipation in PD leaving abundant room for further research addressing this topic.

## Introduction

Parkinson’s disease (PD) is a neurodegenerative disorder in which the loss of dopaminergic neurons in the substantia nigra causes three cardinal motor symptoms: akinesia, rigidity, and tremor. Recently, clinical and scientific attention has shifted to additional nonmotor symptoms that in the past have often passed unheeded.^1^ Of these symptoms, constipation is particularly relevant occurring in up to 66% of all PD-patients, thus showing a higher prevalence than within the general population.^2–5^

Constipation is a syndrome characterized by colonic and anorectal symptoms. The Rome Expert Consensus currently provides the most acknowledged definition of constipation (cf. Table 1).^6^ Underlying causes for constipation in PD are multifaceted. Besides physical weakness, lifestyle risks such as reduced fluid intake may substantially promote its emergence.^7^ Moreover, side effects of medication but also disease-related pathomechanisms have been identified.^8–10^ Regarding the latter, two usually concomitant alterations require distinction: slow intestinal transit and outlet obstruction. Increasing evidence thereby indicates that delayed colonic transit in PD stems from disordered central as well as peripheral parasympathetic system dysregulation.^11^ Additional sacral parasympathetic nuclei and pelvic ganglia affection may foster outlet obstruction. Outlet obstruction, in turn, describes paradoxical contractions or failures of voluntary sphincter relaxation during defecation, which may entail difficulties in rectal evacuation.^12^ An established hypothesis that PD commences in the enteric and progresses to the central nervous system^13,14^ might explain constipation manifesting at early stages of the disease or in some cases even preceding the development of motor symptoms.^15,16^

**Table 1:** Rome IV diagnostic criteria* for functional constipation adapted from (6)

1. Must include 2 or more of the following:**
Straining during more than one-fourth (25%) of defecations
Lumpy or hard stools (BSFS 1-2) more than one-fourth (25%) of defecations
Sensation of incomplete evacuation more than one-fourth (25%) of defecations
Sensation of anorectal obstruction/blockage more than one-fourth (25%) of defecations
Manual maneuvers to facilitate more than one fourth (25%) of defecations (e.g., digital evacuation, support of the pelvic floor)
Fewer than 3 spontaneous bowel movements per week
2. Loose stools are rarely present without the use of laxatives
3. Insufficient criteria for irritable bowel syndrome

*Criteria fulfilled for the last 3 months with symptom onset at least 6 months prior to diagnosis

**For research studies, patients meeting criteria for OIC should not be given a diagnosis of FC because it is difficult to distinguish between opioid side effects and other causes of constipation. However, clinicians recognize that these 2 conditions might overlap

*BSFS* Bristol stool form scale, *OIC* opioid-induced constipation, *FC* functional constipation

All the more alarming, on top of functional impairment, psychosocial distress increases with constipation in PD strongly suggesting negative impact on quality of life.^17–20^ These manifold characteristics of PD-associated constipation doubtlessly highlight an urgent demand for efficacious treatment. Comprehensive and valuable reviews have emerged on the topic of PD-related constipation in recent years.^21–23^ However, little attention has been paid to its management and up-to-date recommendations based on a systematic review analyzing and discussing the effects of a wide range of interventions are lacking. To that end, this work aims at investigating the clinical effectiveness and safety of pharmacological as well as non-pharmacological treatments for constipation in PD.

## Methods

### Eligibility criteria

A systematic literature review was conducted including studies with participants diagnosed with PD and constipation. Because of diverging definitions of constipation in current literature, no restrictions were imposed so that inclusion of studies depended on authors’ definition. Additionally, studies analyzing PD-patients exceeding normal colon transit time (CTT) of <70 h^24^ were likewise included. Studies were only considered if >80% of participants suffered from constipation. All eligible studies investigated the efficacy/effectiveness of therapies for constipation in PD. No obligation of a control group was implied and only experimental and quasi-experimental designs were contemplated, whereas qualitative studies and studies reported in conference abstracts only were excluded. Given the lack of agreement regarding the greatest clinical relevance of outcomes for constipation management, all measures in relation to clinical, bowel movement-related endpoints, satisfaction with treatment as well as colonic and anorectal behavior were considered. Adverse events were assessed as secondary outcome.

### Search strategy and study selection

Three electronic databases (MEDLINE, EMBASE, PsycINFO) were searched until May 2017 using a combination of title/abstract keywords (see Supplementary Data). No time restrictions were applied while only publications in English were considered. A.P. and D.P. selected eligible studies after independently screening titles and abstracts. Full text was retrieved if any uncertainty about eligibility remained.

### Data collection process

For each included study, detailed information was extracted using a standardized data form presented in the supplementary material.

### Quality assessment

Methodological quality was critically appraised by A.P. and D.P. using the Edwards Methods Score.^25^ In this score, higher values represent more elaborate methodology with a maximum of 22 for experimental and 16 for non-experimental studies. Disagreements were resolved via discussion.

### Measures of treatment effect and synthesis of results

For statistically significant results, central tendency along with dispersion measurements were provided for all tested groups pre- and post-intervention. This quantitative data was reported using narrative synthesis.

## Results

**Fig. 1:**
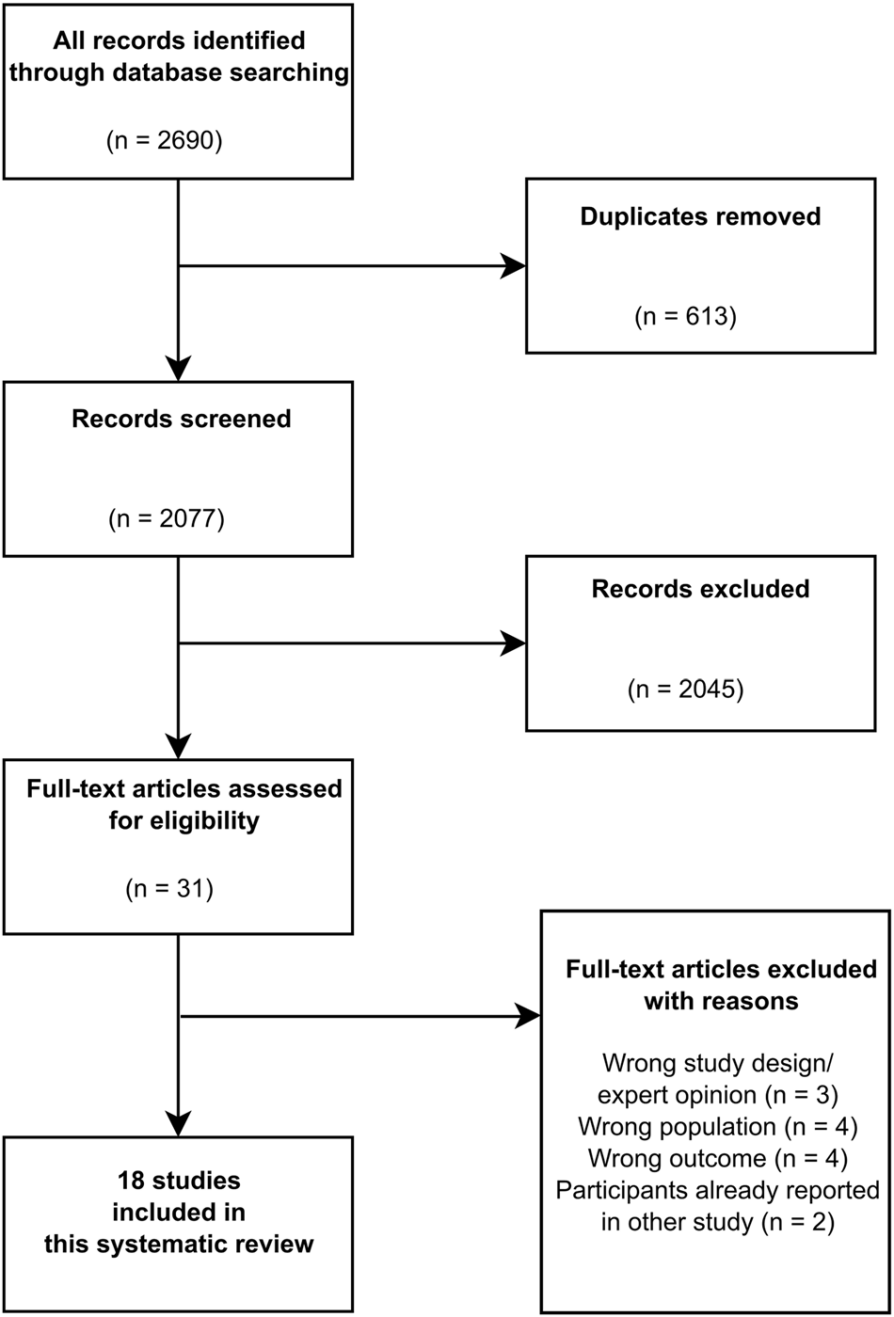
Flow chart of study selection

The search strategy yielded 2690 potential references, of which 613 duplicates were excluded. After screening titles and abstracts, 31 records could be retrieved for full-text evaluation. Hereof, 18 articles were included and 13 excluded according to the eligibility criteria. Figure 1 illustrates the selection process.

### Study characteristics

Seven randomized controlled trials (RCTs) and eleven before-and-after studies fulfilled inclusion criteria of this systematic review. In total, 509 participants were considered for analyses. A tabular overview of their characteristics is provided in Table 2.

**Table 2:** Study characteristics

	Design	Definition of constipation	Intervention
Albanese et al. 2003	BAS (*n* = 10)	Outlet obstruction-type constipation not further specified	Botulinum neurotoxin A injected into the puborectal muscle
Ashraf et al. 1997	RCT (*n* = 7)	<3 BMs/week	Psyllium
Astarloa et al. 1992	BAS (*n* = 19)	<2 BMs/week (considered severe constipation)	Dietetic fiber supplements (wheat, pectin, dimethylpolyoxyhexane-900)
Barichella et al. 2016	RCT (*n* = 120)	Rome III criteria	Multiple probiotic strains and prebiotic fibers
Cadeddu et al. 2005	BAS (*n* = 18)	Outlet obstruction-type constipation characterized by ? Incomplete, prolonged and difficult evacuation with constant use of enemas, laxatives, and manual maneuvers to facilitate bowel movement <3 BMs/week ? Failure to relax perineal floor during straining at physical examination ? Inability to achieve evacuation of barium paste during defecography, with lack of measurable increase in the anorectal angle between rest and attempted evacuation ? Increased activity of the puborectalis muscle at needle EMG ? High pressure levels during straining at anorectal manometry.	Botulinum neurotoxin A injected into the puborectal muscle
Cassani et al. 2011	BAS (*n* = 40)	Rome III criteria	lactobaccilus casei shirota
Chiu et al. 2009	BAS (*n* = 16)	Modified Rome criteria	FMS of thoracic and lumbosacral nerves
Eichhorn and Oertel 2001	BAS (*n* = 8*)	Not specified	Macrogol
Jost and Schimrigk 1997	BAS (*n* = 25)	Delayed CTT of at least 72 h	Cisaprid
Krygowska-Wajs et al. 2016	BAS (*n* = 20)	<3 BMs/week	Deep brain stimulation of the subthalamic nucleus
Liu et al. 2005	BAS (*n* = 7*)	According to a questionnaire on pelvic organ function^89^	Mosapride citrate
McClurg et al. 2016a	RCT (feasibility study, *n* = 32)	Self-reported	Abdominal massage
Ondo et al. 2012	RCT (*n* = 54)	Rome II criteria	Lubiprostone
Parkinson Study Group (2017)	RCT (*n* = 37)	Rome III criteria	Relamorelin
Sakakibara et al. 2005	BAS (*n* = 6*)	Not specified	Dai-Kenchu-To
Sullivan et al. 2006	RCT (*n* = 15)	Rome II criteria	Tegaserod
Tateno et al. 2011	BAS (*n* = 18)	According to a questionnaire on pelvic organ function^89^	Levodopa/carbidopa
Zangaglia et al. 2007	RCT (*n* = 57)	Rome II criteria	Macrogol

*Only PD patients considered, *BAS* before-and-after study, *CTT* colon transit time, *RCT* randomized controlled trial, *FMS* functional magnetic stimulation

### Quality of evidence

The quality scores for studies ranged from 13–20 out of 22 (M ± s.d.: 16.9 ± 2.2) for RCTs and 8–13 out of 16 (M ± s.d.: 10.2 ± 1.6) for quasi-experimental studies.

### Synthesis of results

In what follows results of the included studies will be reported. A summary of the effects on the most frequently used outcome measures can be found in Table 3.

**Table 3:** Clinical effects of the interventions for constipation in Parkinson’s disease

	Stool frequency	Frequency of complete BMs	Stool consistency	Bloating	Straining/difficulty defecating	Feeling of fullness	Pain on defecation	Abdominal pain	Sensation of complete emptying	Colon transit time	Basal sphincter pressure	Maximum squeeze pressure	Anal tone during straining	Rectal tone during straining	Sustained squeeze pressure	Anorectal angle during straining	Abdominal pressure	Rectal sensation	Post-defecation residuals
*Fibers*																			
Ashraf (1997)	+		n.s.		n.s.		n.s.		n.s.	n.s.	n.s.	n.s.			n.s.			n.s.	
Astarloa (1992)	+		+																
*Probiotics*																			
Cassani (2011)	n.s.		+	+				+	+										
*Probiotics and fibers*																			
Barichella (2016)	+	+	+																
*Abdominal massage*																			
McClurg (2016)	n.s.																		
*FMS*																			
Chiu (2009)	+		+	n.s.				n.s.	n.s.	+	+					+			+
*Dopaminergic treatment*																			
Tateno (2011)	n.s.				n.s.					n.s.	n.s.	n.s.	+	n.s.			n.s.	+	+
*STN-DBS*																			
Krygowska-Wajs (2016)				n.s.	+	+													
*Macrogol*																			
Eichhorn and Oertel (2001)	+		+																
Zangaglia (2007)	+		+		+														
*Lubiprostone*																			
Ondo (2012)	+																		
*Cisapride*																			
Jost and Schimrigk (1997)										+									
*Mosapride*																			
Liu (2005)										n.s.	n.s.	n.s.						n.s.	
*Tegaserod*																			
Sullivan (2006)	n.s.		n.s.	n.s.	n.s.			n.s.											
*Relamorelin*																			
Parkinson Study Group (2017)	n.s.	n.s.																	
*Dai-Kenchu-To*																			
Sakakibara (2005)										n.s.			n.s.	n.s.					n.s.
*Botulinum neurotoxin A*																			
Albanese (2003)											n.s.	n.s.	+			+			
Cadeddu (2005)											n.s.	n.s.	+			+			

Significant beneficial effects (+) and non-significant effects (n.s.) of interventions on frequently used outcome measures, *FMS* Functional magnetic stimulation, *STN* Subthalamic nucleus, *DBS* Deep brain Stimulation

### Dietetic interventions

#### Fibers

Two studies scrutinized the effectiveness of soluble fibers for PD-associated constipation. Astarloa and colleagues investigated effects of dietetic supplements (375 mg wheat, 70 mg pectin, 2.5 mg dimethylpolyoxyhexane-900) in 19 PD-patients showing <2 weekly bowel movements (BMs).^26^ Although raw numbers of stool frequency and consistency ratings were not reported, according to the authors severity of constipation improved significantly 2 months after treatment onset with all subjects showing ≥4 BMs/week along with decreased stool consistency. The supplement was well tolerated.

Ashraf et al.^27^ studied effects of psyllium. In a small RCT, three subjects were randomized to 5 mg psyllium BID and four to placebo both administered over eight weeks. Approximate mean values are derived from graphical presentations therefore accuracy may not be claimed. Significant increases in mean stool frequency (2.9 vs. 5.8 BMs/week) and stool weight (400 g vs. 1300 g/week) were reported for psyllium but not for placebo (3.4 vs. 3.5 BMs/week, 400 g vs. 850 g/week). However, neither affected mean CTT significantly. Besides, none of the monthly anorectal manometry parameters were affected. Furthermore, the visual analog scale (VAS) for stool consistency, straining effort, pain on defecation, or completeness of evacuation remained unchanged in both groups. Adverse events were termed mild and similar but not further specified.

#### Probiotics

Cassani et al. studied the effectiveness of probiotics in the treatment of constipation in 40 PD-patients.^28^ A diet rich in fibers and fluid was daily supplemented by 65 ml fermented milk containing 6.5 × 10^9^ colony forming units (CFU) of *lactobaccilus casei shirota* for 5 weeks. While weekly stool frequency and number of days without any BM remained unaltered, significant reduction of days per week at which participants experienced bloating (2.25 ± 2.28 vs. 0.31 ± 0.82; *p* < 0.01), abdominal pain (0.9 ± 1.27 vs. 0.1 ± 0.31, *p* < 0.01) and sensation of incomplete emptying (3.45 ± 2.06 vs. 0.85 ± 1.03, *p* < 0.01) were observed. An increase of days per week in which stools were of normal consistency (1.28 ± 1.41 vs. 3.96 ± 2.02, *p* < 0.01) was also reported. Adverse events were not mentioned.

#### Probiotics and fibers

Barichella et al. investigated the effects of daily intake of 25 × 10^9^ CFU probiotic strains and 7.8 g of fibers contained in 125 ml fermented milk on constipation in PD-patients.^29^ One hundred twenty subjects were randomized to the active or placebo group in a 2:1 ratio. After 4 weeks, the intervention group showed significant increases in the mean number of complete bowel movements (CBMs) (*p* < 0.001) with a mean difference (MD) between groups of 0.7 (95%CI (0.1,1.3), *p* < 0.05). A higher number of participants on supplementation reported ≥3 CBMs (58.8% vs. 37.5%, MD = 2.4, 95% CI [1.1,5.2], *p* < 0.05) than without and a rise by ≥1 CBMs (53.8% vs. 25.0%; MD = 3.5, 95%CI [1.8,8.1], *p* < 0.05) during week 3 and 4. Furthermore, in this period the experimental group presented an increase in stool consistency according to the 7-scaled Bristol stool chart (M: 0.7, 95% CI [0.4,0.9] vs. 0.1, 95% CI [−0.2,0.4], *p* < 0.05). Moreover, participants randomized to the active comparator reported a larger reduction in laxative use (M: −0.8, 95% CI [−1.2,−0.4] vs. M: −0.1, 95%CI [−0.5, 0.2], *p* < 0.05). Participants consuming the milk containing probiotics and prebiotics were more likely to be ‘satisfied’ or ‘very satisfied’ with the intervention (55.0%, vs. 17.5%, MD 5.8, 95% CI [2.3,14.6], *p* < 0.001). Lastly, a higher percentage of participants in the active group stated they were ‘likely’ or ‘very likely’ to continue treatment (56.3% vs. 30.0%, MD 3.0, 95%CI [1.3,6.7], *p* < 0.05). In each group one participant disliked the product whereas one reported abdominal discomfort resulting in withdrawal of the study.

### Physical therapy

#### Abdominal massage

A feasibility study aimed at exploring effects of abdominal massage on PD-related constipation.^30^ Participants received advice on good bowel management and lifestyle but half of all 32 participants were further randomly allocated to an intervention group, in which they/their carers were trained in abdominal massage techniques to be applied daily. Adjusted for baseline symptom scores, there were no significant group differences in the Gastrointestinal Rating Scale, the Neurogenic Bowel Dysfunction Score and the Constipation Score System. Additionally, no significant reduction in stool frequency was demonstrated while significance levels for changes in time spent defecating were not provided. No adverse events were reported.

### Functional magnetic stimulation (FMS)

Chiu et al.^31^ investigated the effects of FMS of thoracic and lumbosacral nerves on colonic and anorectal behavior in 16 constipated PD-patients treated with laxatives and/or enemas. Stimulation was applied 20 minutes BID over three weeks. Mean CTT decreased from 64.9 ± 9.4 h at baseline to 53.6 ± 16.9 h post-intervention (*p* < 0.001). Moreover, widening of the anorectal angle (ARA) during straining (97.9 ± 10.8° to 117.3 ± 14.5°; *p* < 0.001) and between rest and evacuation (6.0 ± 10.9 to 19.3 ± 15.6 degree-difference; *p* < 0.001) were observed, whereas ARA at rest remained unchanged. Furthermore, radiologists’ ratings (1–3) of residual barium amount in the rectum after evacuation indicated improvement (2.63 ± 0.5 to 1.88 ± 0.8; *p* < 0.001). The pelvic floor descent changed from 1.38 ± 2.0 to 2.75 ± 2.2 cm (*p* = 0.002). Alongside, clinical features were assessed using the Knowles–Eccersley–Scott-Symptom Questionnaire (KESS) resulting in reduced mean scores post-FMS (17.5 ± 5.8 to 11.4 ± 5.7; *p* < 0.001) which could be maintained for 12 weeks. Adverse events were not presented.

### Antiparkinsonian therapy

#### Dopaminergic treatment

A before-and-after study evaluated effects of levodopa/carbidopa-therapy (200/20 mg BID) on constipation in 18 de novo PD-patients.^32^ Neither bowel frequency nor defecating difficulties improved significantly after 3 months. Moreover, mean CTT remained unaffected for all colon parts. On average, first sensation during rectal filling diminished from 178.6 ml to 121.3 ml after the treatment period (*p* < 0.05). Simultaneously, mean post-defecation residuals decreased from 142.2 ml to 53.9 ml (*p* < 0.05). Enlargement of spontaneous phasic rectal contraction amplitude, in turn, was not statistically significant. During defecation the amplitude of anal pressure was lessened from 29.7 to −7.1cmH_2_O (*p* < 0.01). Furthermore, the amplitudes of rectal contraction and abdominal straining remained unaltered. According to the authors dopaminergic treatment was well tolerated.

#### Deep brain stimulation of the nucleus subthalamicus

Krygowska–Wajs et al.^33^ assessed gastrointestinal symptoms of 20 PD-patients before and 3 months following surgery for deep brain stimulation of the subthalamic nucleus (STN-DBS). Of these, 19 suffered from constipation and 17 reported defecation difficulties. According to a 5-point assessment (0–4) of symptom severity based on a structured gastrointestinal symptoms questionnaire, the mean score of constipation was 3.28 (Mdn 4, R 0–4) improving to 2.38 (Mdn 3, range 0–4) after STN-DBS (*p* < 0.001). Average severity of defecation difficulty decreased from 2.56 (Mdn 3, R 0–4) to 1.29 (Mdn 1, R 0–3; *p* < 0.001) and feeling of fullness improved from 1.52 (Mdn 0, R 0–4) to 1.05 (Mdn 0, R 0–4; *p* < 0.001). Scores for bloating did not change significantly. Adverse events were not reported.

### Laxatives

#### Macrogol

Two studies investigated effects of isoosmotic macrogol electrolyte solution on PD-related constipation. A double-blind RCT assessed efficacy and safety of macrogol (7.3–21.9 g BID) in 57 patients.^34^ Authors defined treatment efficacy as complete relief of the predominant symptom or a marked improvement of at least two clinical indicators: (i) stool frequency, (ii) straining, (iii) stool consistency, or (iv) rescue therapy with rectal laxatives. Accordingly, responder rates in the active group significantly outnumbered those in the placebo group at four (78.3% vs. 25.0%, *p* < 0.001) and at 8 weeks (80.0% vs. 30.4%, *p* < 0.05). Stool frequency was higher (*p* < 0.05) with macrogol (baseline: 1.9 ± 0.56; week 4: 5.7 ± 2.3; week 8: 6.6 ± 2.7, both *p* < 0.001) compared to placebo (baseline 2.0 ± 0.61; week 4: 3.4 ± 1.7, *p* < 0.001; week 8: 3.7 ± 1.9, *p* < 0.05). Additionally, differences in stool consistency favoring macrogol were reported (week 4: *p* < 0.05; week 8: *p* < 0.001). Responder rates for straining revealed significant effects in both groups at four weeks (macrogol 18.5 ± 24; placebo 30.7 ± 29.7; both *p* < 0.05), whereas at 8 weeks no significant effect was traceable between the two groups. Two participants on placebo but none on macrogol used rescue treatment with rectal laxatives. Two patients in the intervention group discontinued due to nausea and diarrhea respectively.

As part of a before-and-after study, eight PD-patients suffering from constipation were treated with a solution of 13–39 g macrogol.^35^ Baseline information on stool frequency was imprecise ranging from one BM every 14 days to twice per week. However, mean stool frequency increased to 4 BMs/week after 9–21 weeks of treatment with a minimum of 3 and a maximum of 7 BMs/week. All participants reported moderate or marked improvement in stool consistency and marked improvement in ease of defecation and global impression change. No adverse events were indicated.

#### Lubiprostone

In a double-blind RCT efficacy and safety of lubiprostone were assessed in 54 PD-patients.^36^ The dosage in the active group was titrated up to 48 µg daily, which participants were allowed to reduce to 24 µg, if not tolerated. At 4 weeks, 16 of 25 subjects (64.0%) assigned to lubiprostone reported a marked or very marked clinical global improvement relative to five of 27 subjects (18.5%) receiving placebo (*p* = 0.001). Additionally, increased stool frequencies (lubiprostone 0.75 ± 0.80 to 0.97 ± 0.88 BMs/day, placebo 0.84 ± 0.76 to 0.83 ± 0.76, *p* = 0.001), higher scores on the VAS (lubiprostone 51.4 ± 8.5 to 71.2 ± 16.6, placebo 50.7 ± 5.9 to 56.8 ± 13.0; *p* < 0.001) as well as better scores in the bowel movement review questionnaire (lubiprostone 13.3 ± 4.91 to 6.6 ± 1.11, placebo 13.4 ± 4.8 to 10.2 ± 6.5; *p* < 0.05) were reported. Loose stools were more common in the intervention group (12 [48.0%]) vs. 1 case [3.7%]) but mostly mild and self-limiting.

#### Cisapride

Jost and Schimrigk examined the effectiveness of cisapride in 25 PD-patients with delayed CTT.^37^ After 1 week of 5 mg cisapride BID, mean CTT diminished from 131 h to 81 h (*p* < 0.01). Long-term results of cisapride 20 mg daily, however, showed a weakened effect at 6 (99 h, *p* < 0.01) and 12 months (118 h, *p* < 0.01) Significance levels of changes in symptom burden were not provided. Cisapride was well-tolerated without serious side effects.

#### Mosapride

In a before-and-after study, Liu et al.^38^ evaluated mosapride effects on constipation in 14 subjects including 7 PD-patients. In the PD-population, CTT remained unaltered for any colon part. Results of anorectal videomanometry were not specifically reported for PD-patients. No adverse events were observed.

#### Tegaserod

In a double-blind RCT, Sullivan et al. assessed the efficacy of tegaserod for PD-related constipation in 15 subjects.^39^ Participants were randomly allocated to receive either 6 mg of tegaserod BID or placebo. After 4 weeks, constipation was re-evaluated in both groups applying the Subject’s Global Assessment of abdominal discomfort/pain, symptom relief, bowel habits, satisfaction with bowel habits, bloating, straining, stool frequency and consistency. Thereby, none of the measures differed significantly from baseline and no side effects occurred.

#### Relamorelin

The Parkinson study group conducted a double-blind RCT to investigate treatment effects of 100 µg relamorelin on PD-associated constipation.^40^ Following a 4-day baseline period in which participants received placebo, either relamorelin or placebo was administered subcutaneously once daily over two weeks. No significant group differences were detected with regard to BMs/week, spontaneous BMs/week, complete BMs/week, complete spontaneous/week and days without BMs. However, the trial did not meet the recruitment goal of 56 participants as only 18 of 37 subjects involved in the baseline period were randomized into phase 2 primarily by reason of too high stool frequencies according to the study protocol. No serious adverse events were reported.

#### Herbal medicine

Sakakibara et al. investigated the effects of 15 g Dai-Kenchu-To (50% ginger, 30% ginseng, 20% Zanthoxylum) TDS on constipation in 10 subjects including 6 PD-patients.^41^ After 12-week administration CTT, rectal pressure at rest and during defecation, anal pressure as well as post-defecation residuals remained unchanged in the PD-population. Except for bitter taste Dai-Kenchu-To was well-tolerated.

#### Botulinum neurotoxin

Two before-and-after studies examined the benefit of botulinum neurotoxin A (BTX) in the treatment of outlet obstruction constipation in PD-patients. In Albanese et al.’s study, 10 patients suffering from isolated or prominent outlet obstruction received an injection of 100U BTX into the puborectal muscle under transrectal ultrasonographic guidance.^42^ The mean anal tone during straining decreased from 97.4 ± 19.6 mm Hg to 40.7 ± 11.5 mm Hg and to 38.2 ± 10.4 mm Hg (both *p* < 0.001) at 2 months post-injection. Resting anal tone and maximum contraction remained unchanged. Furthermore, the anorectal angle during straining augmented from 99 ± 7.9° to 122.2 ± 15° at two months (*p* < 0.001) while at rest it remained unaltered.

In a similar study, Cadeddu et al. tested the same intervention in 18 PD-patients with outlet obstruction constipation.^43^ While after 1 month symptomatic improvement was traceable in 8 participants, at 2 months the number increased to 10 participants. Compared to baseline, mean resting pressure and maximum voluntary contractions did not differ at both time points. However, anorectal manometry demonstrated a decreased tone during straining at 1-month (96.2 ± 17.1 to 45.9 ± 16.2 mmHg) and 2-month evaluation (56.1 ± 10.7 mmHg, both *p* < 0.001). ARA did not change significantly while during straining it decreased from 99.1 ± 8.4° to 121.7 ± 12.7° (*p* < 0.001). Ten patients without satisfactory benefit after the first intervention were re-treated with 200U resulting in symptomatic improvement in four participants at 2 months. In this cohort, ARA during straining increased from 100.1 ± 7.2° to 119 ± 8° (*p* < 0.001), whereas resting anal pressure and voluntary contraction remained unchanged. Pressure during straining was reduced from 90.7 ± 21.6 to 61.2 ± 17.4 mmHg at 1-month (*p* < 0.05) and 59.7 ± 19.1 mm Hg at 2-month assessment (*p* < 0.05) being significantly lower in relation to resting anal pressure. At 4 months, six subjects experienced symptomatic recurrence and were re-injected 200–300U. In these participants pressure during straining diminished from 89.7 ± 30.4 to 58.7 ± 15.0 mm Hg at one-month evaluation (*p* < 0.05) and to 56.4 ± 16.0 mm Hg at 2-month evaluation (*p* < 0.05) without significantly changing resting pressure or maximum contraction. ARA during straining increased from 99.6 ± 8° to 121.9 ± 12.1° (*p* < 0.05).

While Albanese and colleagues did not disclose adverse events, Cadeddu et al. stated that no side effects occurred.

## Discussion

Constipation as one of the most frequent symptoms in PD constitutes considerable hardship on the emotional, psychological, and social well-being of patients.^20^ To reduce symptom burden, timely and effective treatment is thus essential. However, this systematic literature review provides five crucial results complicating structured recommendations for constipation management in PD: (i) Only few clinical studies address the effectiveness and safety of different treatment options, (ii) many studies were prone to bias, (iii) most studies differentiate insufficiently between pathomechanisms of constipation, (iv) the plethora of validated and non-validated outcome measures hampers comparison and discourages from the conduction of a meta-analysis, (v) no head-to-head trial drew direct comparisons between therapies. Yet, some conclusions on potential therapies may be drawn from the available literature which are discussed in what follows under six subheadings: dietetic interventions, physical therapy, antiparkinsonian therapy, laxatives, herbal medicine and local botulinum toxin treatment.

### Dietetic interventions

A high quality study by Barichella et al.^29^ offered first evidence for the efficacy of fibers combined with probiotics for PD-associated constipation. However, there is only limited data available on separate effects of the two components.

Active principles of fibers comprise two mechanisms.^44^ First, indigestible fibers increase stool volume, thereby enhancing water-binding capacity and stimulating microbial growth and gas production. Furthermore, they intensify colonic motility through mechanical actions caused by the stool but also through colokinetic products resulting from fiber fermentation. Nevertheless, little evidence in favor or against the use of dietary soluble fiber supplements in the treatment of PD-related constipation is provided by the two identified studies.^26,27^ This might be ascribed to small numbers of participants and limited methodological quality. However, ideas about beneficial effects in PD may not be far-fetched considering moderate effectiveness in the management of chronic idiopathic constipation (CIC).^45^ A therapeutic approach with fibers is yet appealing given their manageable adverse events and low cost profile, encouraging further research in this field.

Probiotics are live or attenuated microorganisms with multiple putative mechanisms of action that are attributed ameliorating effects on constipation.^46^ In the study by Cassani et al.^28^, stool frequency of PD-patients on a diet rich in fibers and fluid remained unaltered after *Lactobacillus casei shirota* administration. In contrast, stool consistency along with subjective measures such as bloating, pain and sensation of incomplete emptying improved significantly. However, cautious interpretation is advised not only because of small sample size, but especially as results of the before-and-after design are inestimably susceptible to temporal trends, bias and confounders. Nevertheless, in a population with CIC two RCTs revealed moderate effects of *Lactobacillus casei shirota* on stool consistency and frequency with one of them suggesting additional favorable effects on CTT.^47,48^ In view of these findings, experimental studies are warranted to investigate possible benefits of probiotics on constipation in PD.

In summary, this systematic review may not render unambiguous evidence supporting the use of dietetic interventions for PD-associated constipation. However, clinical experience suggests simple lifestyle modifications including increased fiber and fluid intake being reasonable and beneficial prior but also supplementary to pharmacological treatment.^9,49–51^ In conformity with these opinions, this approach may be discussed with patients according to principles of shared decision-making.

### Physical therapy

Two studies evaluated the effects of two domains of physical therapy on constipation in PD: abdominal massage and FMS. A few studies showed that abdominal massage alleviates CIC effectively through stimulating peristalsis, decreasing CTT and hence increasing stool frequency.^52–54^ However, these results stem from heterogeneous research of limited methodological quality. In PD-patients, research by McClurg et al.^30^ failed to demonstrate significant clinical improvement in, e.g., stool frequency. Yet, it must be stressed that primary objectives of this small pilot study were testing recruitment, retention and the appropriateness of the intervention and outcome measures. A positive effect would be appealing insofar as performed by the patient or carer it is inexpensive and additionally free of harmful side effects. Alike most complex interventions, problems could relate to difficulties of standardizing its delivery to ensure quality.

The exact mechanism of action of FMS remains unclear. Several studies suggested sacral nerve stimulation improving outcomes of anorectal manometry in CIC while results regarding CTT were conflicting.^55,56^ In the context of PD, patients benefitted mildly from adjuvant thoracic and lumbosacral FMS in their colonic and anorectal behavior.^31^ The clinical improvement shown in the KESS, may be questionable given the debatable psychometric properties of this questionnaire.^57,58^

In summary, there is insufficient evidence to support or refute application of physical therapy in PD-associated constipation. However, as measures are generally well tolerated and side effects are scarce there is room for further research.

### Antiparkinsonian therapy

Abundant evidence indicates reduction in motor symptom burden and better long-term motor outcomes with timely and tailored antiparkinsonian treatment initiation.^59^ In contrast, repercussions of antiparkinsonian medication for constipation remain subject of controversial debate. While some authors attribute constipation to dopaminergic treatment,^60,61^ Tateno et al.^32^ contrarily suggested that levodopa might ameliorate outlet obstruction constipation in treatment-naive patients. Despite significant changes in anorectal manometry, cautious interpretation is advised for several reasons. First, to date, it remains unclear whether statistical differences in manometrical measures necessarily imply notable clinical improvement. Standard values strongly depend upon technical and patient-specific parameters,^62^ which was not addressed by the authors. Second, the rather small and restricted population and the well-known flaws of the before-and-after design may limit their findings.

Similar limitations may likewise apply to the small before-and-after study investigating STN-DBS,^33^ which is currently primarily recommended to patients suffering from long-term motor complications. However, in this study DBS significantly improved constipation including difficulty with defecation 3 months following lead implantation. These results may corroborate earlier research reporting not only decreased Unified Parkinson Disease Rating Scale scores for constipation^63,64^ but also improvement of gastrointestinal symptoms, possibly through modulation mediated by central nervous regulation by STN-DBS.^65,66^ For a full picture, additional studies evaluating this invasive intervention on outcomes related to constipation are needed.

Above the aforementioned necessity of tailored antiparkinsonian therapies for motor symptoms, the poor evidence neither allows to substantiate nor to disprove potential benefits of levodopa and STN-DBS for constipation. However, clinicians may bear a Cochrane review in mind, which indicated constipation being less frequent with levodopa than with dopamine agonists.^67^ In other words, despite the lack of direct data on effects of dopamine agonists on constipation, one may consider the strategy of switching to levodopa if feasible. Yet, more research is required to clearly understand associations between antiparkinsonian therapy and constipation.

### Laxatives

Macrogol is an osmotic laxative, which increases the amount of fluids in the bowel thereby softening the stool. Two studies provided an indication for the effectiveness and safety of macrogol in PD-associated constipation.^34,35^ Stool frequency and consistency improved significantly in both studies and superiority over placebo was demonstrated by Zangaglia et al. Remarkably, results are prone to bias as both studies analyzed small sample sizes with the RCT incurring high attrition consecutively being underpowered. Nevertheless, the clinical findings corroborated results of a meta-analysis including a heterogeneous population suffering from chronic constipation, in which macrogol proved being safe and additionally more effective than lactulose.^68^ Another study also found macrogol being superior to tegaserod.^69^ Substantiated by these findings, further research evaluating the effects of macrogol in PD is recommended.

Lubiprostone activates chloride channels on the apical surface of gastrointestinal epithelial cells enhancing chloride-rich fluid secretion. It therefore softens the stool and increases motility. Congruent with findings of studies analyzing patients suffering from CIC, Ondo et al. reported superiority of lubiprostone over placebo in the improvement of clinical outcomes.^36,70^ With current knowledge indicating a manageable side-effects profile, results for the treatment of PD-associated constipation with lubiprostone appear promising. However, one should be mindful that while lubiprostone is approved for the use in chronic constipation in the US, Japan, Switzerland and the UK, to date it is unavailable in numerous other countries. Besides, lubiprostone constitutes a relatively expensive treatment option, requiring future head-to-head trials to investigate not only its efficacy and safety but also its cost-effectiveness. In the best case, evaluation periods assessed should exceed four weeks thereby facilitating inference for clinical long-term use.

Apart from dopaminergic influences, neurotransmitters such as acetylcholine and serotonin significantly impact on nonmotor symptoms in PD.^71^ Anticholinergics were among the first drugs available for PD therapy and for obvious reasons are contraindicated in PD-patients suffering from constipation. The 5-HT_4_ agonists cisapride, mosapride and tegaserod, in turn, may be considered a promising starting point for a tailored therapy in PD-associated constipation stimulating gastrointestinal motility.^72^ Indeed, it was postulated that cisapride may be effective in the short-term treatment but long-term results were discouraging in a PD-population.^37^ Likewise treatment with mosapride did not entail significant changes in CTT in 7 PD-patients^38^ whereas clinical effects of tegaserod on clinical parameters in a small RCT were not significant.^39^ Correspondingly, studies in a general population with CIC failed to demonstrate clear benefits of both cisapride and tegaserod.^69,73^ However, there are few but supporting studies demonstrating that mosapride may be effective for constipation in mixed and non-PD-populations.^38,74,75^ Nonetheless, all three 5-HT_4_ agonists were not globally granted market authorization. Cisparide and tegaserod were even withdrawn from some markets or granted for restricted indications only owed to their increased risk of fatal cardiac arrhythmias. Since chronic constipation is not associated with a high mortality risk, a cautious risk-benefit-analysis is pivotal. Even though in the presented studies both substances were well-tolerated, the elsewhere reported potential undesirable consequences outweigh possible desirable effects, strongly advocating against cisapride and tegaserod in PD-associated constipation. Despite mosapride not showing comparable effects on cardiovascular function,^72^ its effectiveness in PD-associated constipation has not been evidenced.

Studies indicated that the synthetic ghrelin agonist relamorelin may be effective in increasing stool frequency and accelerating colonic transit.^76^ However, so far marketing authorization for relamorelin in constipation has not been granted. Recently, the Parkinson study group could not detect significant clinical benefits for relamorelin in PD-associated constipation which may, however, be attributed to the failure of meeting the recruitment target.^40^ Therefore, further studies need to be carried out in order to establish whether relamorelin is effective and safe in the management of constipation in PD. In this case the use as rescue medication appears most obvious due to the subcutaneous route of application.

In summary, there is a substantial research gap regarding the use of available and widely used laxatives for constipation in PD. Quality of evidence for the use of lubiprostone and macrogol in PD-associated constipation is comparable. Due to choice of different outcome measures and selective reporting, superiority of one of either cannot be deduced. Nevertheless, according to the sparse literature available to date and in light of economic considerations macrogol may be given preference over lubiprostone.

### Herbal medicine

Small studies scrutinizing the effects of the Japanese medicine Dai-Kenchu-to on constipation provided inconclusive evidence.^77–79^ Moreover, the study by Sakakibara et al. did not contribute supporting evidence for its effectiveness in a cohort of constipated PD-patients.^41^

### Botulinum neurotoxin

BTX blocks nerve impulses entailing flaccid muscle paralysis. Small studies demonstrating the effectiveness and safety of injections of BTX in the puborectal muscle for the treatment of outlet obstruction of different etiologies were corroborated by two studies in PD-populations.^42,43,80–88^ Although injections might improve anorectal manometry parameters suggesting anal sphincter relaxation, patient-reported outcomes were so far disregarded and need acknowledgement by future studies. The initial relatively low responder rate in the study by Cadeddu et al. might be attributable to underdosing or inaccurate injection. Thus, BTX may be considered for treatment of isolated or prominent outlet constipation in PD, however, placebo-controlled studies with long-term follow-up are warranted to ascertain efficacy and duration of effects beyond 2 months.

### Limitations

This systematic review was limited to certain study designs published in peer-reviewed journals with language restrictions possibly leading to publication and language bias. This might imply an overestimation of effects. Furthermore, no author was contacted for information unavailable in the publications. Lastly, acknowledging the manifold definitions of constipation and to not depreciate patients’ perceptions, we left diagnostic criteria to authors’ discretion. However, this might occasion a heterogeneous population complicating clinical application of results.

## Conclusion

The strength of evidence for the effectiveness of the presented treatment options is impacted by small, heterogeneous trials and their restricted quality. The current state of research is therefore insufficient to provide clear recommendations on a first-line treatment of PD-associated constipation. However, lifestyle and dietetic adjustments may promote constipation relief, whereas macrogol and lubiprostone may be contemplated as medical therapies. In the specific case of isolated or prominent outlet obstruction constipation, the injection of botulinum neurotoxin A possibly improves symptoms. In the future, studies assessing standardized and clinically relevant outcomes would be conducive particularly targeting head-to-head comparisons in order to identify the most effective treatments with tolerable side effect profiles.

## Electronic supplementary material

Supplemental Material(DOCX 70 kb)
