# Supplementary material for: Management of constipation in patients with Parkinson’s disease
Source: NPJ Parkinsons Dis. 2018 Mar 16;4:6. doi: 10.1038/s41531-018-0042-8 (PMC5856748; doi:10.1038/s41531-018-0042-8)
Supplement: Supplementary file 1 — Supplemental Material(DOCX 70 kb) [file 41531_2018_42_MOESM1_ESM.docx]

# Data supplement

## Search strategy

**Medline (OVID) search strategy**

1 Parkinson disease/

2 parkinson$.ti,ab.

3 1 or 2

4 constipation/

5 (constipat$ or obstipat$ or coprostasis).ti,ab.

6 4 or 5

7 3 and 6

8 limit 7 to human

**Embase (OVID) search strategy**

1 Parkinson disease/

2 parkinson$.ti,ab.

3 1 or 2

4 constipation/

5 (constipat$ or obstipat$ or coprostasis).ti,ab.

6 4 or 5

7 3 and 6

8 limit 7 to human

**PsycInfo (OVID) search strategy**

1 exp Parkinson's Disease/

2 parkinson$.ti,ab.

3 1 or 2

4 exp Constipation/

5 (constipat$ or obstipat$ or coprostasis).ti,ab.

6 4 or 5

7 3 and 6

8 limit 7 to human

## Data extraction spreadsheet

| **First author (year)** | **Journal** | **Country** | **Design** | **Population** | **Definition of constipation** | **Intervention** | **Main results** | **Edwards Score** |
| --- | --- | --- | --- | --- | --- | --- | --- | --- |
| Albanese  (2003) | Am J Gastroenterol | Italy | Before-and-after study | 10 PD-patients with isolated or prominent outlet obstruction constipation | Outlet obstruction-type constipation not further specified | Botulinum neurotoxin A  (Injection of 100 U into the puborectal muscle under transrectal ultrasonographic guidance) | 1°: Evaluation by means of anorectal manometry, defecography, and electrography (baseline, at 1 and 2 months post-treatment):  mean anal tone during straining decreased from 97.4±19.6 mmHg at baseline to 40.7±11.5 mmHg (p=0.00001) and to 38.2±10.4 mmHg (p=0.00001) at one month and 2 months post-treatment respectively. Resting anal tone (59.2±21.2 vs. 49.4±13.9 vs. 56.8±20.9 mmHg) and maximum voluntary contraction (60.1±30.5 vs. 52.4±30.3 vs. 61.7±22.5) did not change significantly. The anorectal angle during straining augmented from an initial mean of 99±7.9 degrees to 122.2±15 degrees after two months (p=0.0004) while the anorectal angle at rest did not change (115.5±7.5 to114±9.1)  2°: not reported | 11/16 |
| Ashraf (1997) | Mov. Disord. | USA | Randomized, single-blind, placebo-controlled trial | 7-PD patients with constipation | <3 BMs/week | Psyllium 5.1 mg BID | 1°: Significant increase in both stool frequency and stool weight in active group but not in placebo group, psyllium did not have a significant effect on any parameters of anorectal manometry, VAS scores for stool consistency, straining effort, pain on defecation, or completeness of evacuation remained unchanged in both groups  2°: no significant differences in AE, symptoms were mild, no withdrawal | 18/22 |
| Astarloa (1992) | Clinic. Neuropharmacol | Spain | Before-and-after study | 19 PD-patients with severe constipation | <2 BMs/week (considered severe constipation) | Dietetic fiber supplements (375mg wheat, 70mg pectin, and 2.5mg dimethylpolyoxyhexane-900) before L-Dopa intake | 1°: severity of constipation improved significantly (p<0.001), at least 4 weekly bowel movements, consistency of feces decreased  2°: 1 patient withdrew due to flatulence | 8/16 |
| Barichella (2016) | Neurol. | Italy | Randomized, double-blind, placebo-controlled trial | 120 PD-patients with constipation | Rome III criteria | Fermented milk, containing 25x109 CFU multiple probiotic strains (Streptococcus salivarius subsp thermophilus, Enterococcus faecium, Lactobacillus rhamnosus GG, Lactobacillus acidophilus, Lactobacillus plantarum, Lactobacillus paracasei, Lactobacillus delbrueckii subsp bulgaricus, and Bifidobacterium breve and animalis subsp lactis) and 7.8g prebiotic fiber | Treatment resulted in increase of the  number of CBMs (p <0.001), while the use of placebo  resulted in a nonsignificant change (p=0.76). At 4 weeks, the intervention group showed a mean increase in CBMs of 1.2 (95%CI [0.8,1.6]) compared with 0.1 (95%CI [20.4,0.6]) in the control group (mean difference 1.1, 95%CI [0.4,1.8]; p=0.002). A higher number of participants in the intervention group reported ≥3 CBMs than in the placebo group ((58.8% vs 37.5%; p=0.030)) and an increase by ≥1 CBMs (53.8% vs 25.0%; p=0.004;) during weeks 3 and 4. Furthermore, in this period the experimental group presented a higher increase in the mean number of total BMs (0.7, 95%CI [0.3,1.0] vs. 0, 95%CI [-0.2,0.4]; p=0.023) and in mean stool consistency according to the Bristol stool chart (0.7, 95%CI [0.4,0.9] vs. 0.1, 95%CI [-0.2,0.4]; p=0.018). Participants in the intervention group reported a larger reduction in the use of laxatives (-0.8, 95%CI [-1.2,-0.4] vs. -0.1, CI95% [-0.5,0.2]; p=0.018). Participants in the intervention group were more likely to be ‘satisfied’ or ‘very satisfied’ with the intervention 55.0%, 95%CI [40.0,73.8] vs. 17.5% 95%CI [7.0,36.1]; p<0.001). Participants in the intervention group were more likely or likely to continue the intervention: 56.3%, 95%CI [41.0,75.3] vs. 30.0 95%CI [15.5, 52.4],; p=0.008).  2°: In each group 1 participant disliked the product and 1 reported abdominal discomfort | 20/22 |
| Cadeddu (2005) | Aliment. Pharmacol. Ther. | Italy | Before-and-after study | 18 PD-patients with outlet type constipation | outlet type constipation characterized by   - Incomplete, prolonged and difficult evacuation with constant use of enemas, laxatives, and manual maneuvers to facilitate bowel movement - <3 evacuations/   week   - Failure to relax perineal floor during straining at physical examination - Inability to achieve evacuation of barium paste during defecography, with lack of measurable increase in the anorectal angle between rest and attempted evacuation - Increased activity of the puborectalis muscle at electromyography performed with needle electrode - High pressure levels during straining at anorectal manometry. | Botulinum neurotoxin A  (Injection of 100 U into the puborectal muscle under transrectal ultrasonographic guidance)  If symptoms persisted after 2-month evaluation re-treatment considered with 200-300 U | Evaluation by means of clinical features and anorectal manometry and defecography (baseline, at 1 and 2 months post-treatment):  A symptomatic improvement reported by 8 subjects at 1 month (incomplete evacuation disappeared in 5 and reduced in 3) (p=0.002) and 10 at 2 months, (p=0.0003); mean anal tone during straining decreased from 96.2±17.1 mmHg at baseline to 45.9±16.2 mmHg (p=0.00001) and to 56.1±10.7 mmHg (p=0.00001) at one month and 2 months post-treatment respectively. Resting anal tone (60.1±21.4 vs. 51.4±16.1 vs. 56.8±22.1 mmHg) and maximum voluntary contraction (61.4±31.2 vs. 54.6±29.7 vs. 60.7±22.8) did not change significantly. The anorectal angle during straining augmented from an initial mean of 99.1±8.4 degrees to 121.7±12.7 degrees after two months (p=0.00001) while the anorectal angle at rest did not change (116.1±8.0 to113.7±9.2). Rescue medication at 2 months for 8 symptomatic participants:  Symptomatic improvement in subjects, mean anal tone during straining decreased from 90.7±21.6 mmHg to 61.2±17.4 mmHg (p=0.009) and to 59.7±19.1 mmHg (p=0.008) at one month and 2 months post-treatment respectively  Resting anal tone and maximum voluntary contraction did not change significantly. The anorectal angle during straining increased from an initial mean of 100.1±7.2 degrees to 119±8 degrees after two months (p=0.0002)  At 4 months, 6 patients suffered symptomatic recurrence and were re-treated with 200-300 U:  Resting anal pressure and voluntary pressure unchanged  Pressure during straining was reduced from 89.7±30.4 to 58.7±15 mmHg (p=0.04) at 1-month and to 56.4±16 mmHg at 2-month evaluation, aorectal angle during straining increased from 99.6±8 degrees to 121.9±12.1 degrees (p=0.003)  2°: no side effects | 10/16 |
| Cassani (2011) | Minerva Gastroenterol. Dietol. | Italy | Before-and-after study | 40 PD-patients with constipation | Rome III criteria | 65ml fermented milk containing 6.5x109 CFU of lactobaccilus casei shirota daily | 1°: comparison baseline and week 6 for days per week with normal stool consistency (1.28±1.41 vs. 3.96±2.02, p<0.01), bloating (2.25±2.28 vs. 0.31±0.82, p<0.01), abdominal pain (0.9±1.27 vs. 0.1±0.31, p<0.01), sensation of incomplete emptying (3.45±2.06 vs. 0.85±1.03, p<0.01), number of BMs (4.75±2.59 vs. 4.93±1.85, NS), days without any BM (2.85±1.64 vs. 2.13±1.71, NS)  2°: AE not reported | 10/16 |
| Chiu (2009) | J Rehabil Med | Taiwan | Before-and-after study | 16 PD-patients with constipation | ≤2 BMs/week, the use of laxatives or enema more than once a week or digital evacuation of faeces on all occasions” | FMS of thoracic and lumbosacral nerves 20 minutes twice a day over three weeks | 1°:baseline means±SD vs. post-FMS:  Rest ARA 94.0±9.9° vs. 100.8±12.2° (NS), evacuation ARA 97.9±10.8° vs. 117.3±14.5° (P<0.001), degree difference 6.0±10.9° vs. 19.3±15.6° (P<0.001), pelvic floor descent 1.38±2.0 vs. 2.75±.2cm (p<0.002), residual barium amount score 2.63±0.5 vs. 1.88±0.8 (P<0.001), CTT 64.9±9.4 vs. 53.6±16.9 (P<0.001), KESS score 17.5±5.8 vs. 11.4±5.7 (P<0.001)  2°: AE not reported. | 13/16 |
| Eichhorn & Oertel (2001) | Mov. Disord. | Germany | Before-and-after study | 8 PD-patients with sever constipation for 1 year  (2 MSA-patients disregarded) | Not specified | Macrogol 13-39g, (maintenance dose 26g/day to 13g/3rd day) | 1°: In all PD patients stool frequency at baseline ranged from one bowel movement every second week to twice per week and increased to a mean (SD) of 4 (1.22) per day after a treatment period of 9 to 21 weeks. 1 PD patient reported a moderate improvement in stool consistency, 7 PD marked. All marked improvement in ease of defecation and global impression change. Dose could be reduced  2°: No adverse effects reported | 9/16 |
| Jost & Schimrigk (1997) | Mov. Disord. | Germany | Before-and-after study | 25 PD-patients with delayed CTT | Delayed CTT of at least 72h | Cisaprid 5 mg BID for 7 days, then 10 mg BID for one year | 1°: Mean CTT decreased from 131h at baseline to 81h at 1 week, 99 h at 6 months and 118 at 1 year (all p<0.01).  <3 BMs/week: 21 vs. 6 vs. 8 vs. 15  Hard stools: 18 vs. 9 vs. 11 vs. 14  Difficult defecation: 22 vs. 11 vs. 15 vs. 18  Tenesmus: 15 vs. 7 vs. 9 vs. 12  Sensation of incomplete evacuation: 18 vs. 13. vs. 14. vs. 17  2°: No central side effects | 10/16 |
| Krygowska-Wajs (2016) | Parkinsonism Relat Disord | Poland | Before-and-after study | 20 PD-patients (including 19 with reported constipation and 17 with difficulty with defecation) | <3 BMs/week  (difficulty with defecation=difficulty with the act of defecation, straining and/or sense of incomplete evacuation) | Subthalamic deep brain stimulation | 1°: 5-point assessment (0-4) of the severity of symptoms:  mean score of constipation of 3.28 (median 4, range 0-4) significantly improved to 2.38 (median 3, range 0-4) after 3 months (p<0.001), severity of difficulty with defecation decreased from a mean score of 2.56 (median 3, range 0-4) to 1.29 (median 1, range 0-3; p<0.001). Feeling of fullness scored with a mean of 1.52 (median 0, range 0-4) significantly improving to 1.05 (median 0, range 0-4; p<0.001). Bloating did not change significantly with means of 1.32 at baseline (median 1, range 0-4) and 1.33 post-intervention (median 1, range 0-4)  2°: No side effects reported | 12/16 |
| Liu (2005) | Mov. Disord. | Japan | Before-and-after study | 7 PD-patients with constipation  (7 MSA-patients disregarded) | According to a questionnaire on pelvic organ function (Sakakibara et al., 2001) | 15 mg/day of mosapride citrate | 1°: at 3 months no significant changes in CTT for total colon (107.7 to 75.2 h), right colon (27.8 to 21.4 h), left colon (32.6 to 26.4 h), rectosigmoid (39.2 to 31.2 h), results of anorectal manometry not reported for PD-population  2°: 1 withdrawal due to epigastric discomfort | 8/16 |
| McClurg (2016a) | Age Ageing | UK | Randomized controlled trial  (feasibility study) | 32 PD patients with self-reported constipation | Self-reported | Abdominal massage | 1°: At 10 weeks, adjusted for baseline symptom scores there was no significance between group difference in the Gastrointestinal Rating Scale (−1.81; 95%CI −7.01, 3.39; p=0.477), the Neurogenic Bowel Dysfunction Score (−0.22; 95%CI −2.79, 2.34, p=0.859) and the Constipation Score System (0.82; 95%CI −2.02, 3.66; p=0.555). A bowel diary did not demonstrate any significant reduction in the frequency of BMs, but a decrease in time spent defecating from 10±2.5min to 4.5±1.4min in the intervention group and from 5±2.4min to 4±1.5min in the control group.  2°: no AE reported | 17/22 |
| Ondo (2012) | Neurol. | USA | Randomized, double-blind, placebo-controlled trial | 54 PD patients with constipation | Rome II criteria | Lubiprostone 24ug/day for 7 days in the morning, then increased to 2 intakes daily  (if not tolerated decrease to 1) | 1°: Of the 25 participants assigned to lubiprostone 1 reported a mildly worse change, 3 no change, 6 a mildly improved , 9 much improved and 7 very much improved change. Whereas in the placebo group 4 reported a mildly worse change, 12 no change, 6 a mildly improved , 3 much improved and 2 very much improved change (p=0.001), increased stool frequencies (lubiprostone 0.75±0.80 to 0.97±0.88 BM/day, placebo 0.84±0.76 to 0.83±0.76; p=0.001), higher scores on the VAS (lubiprostone 51.4±8.5 to 71.2±16.6, placebo 50.7±5.9 to 56.8±13.0; p<0.001), better scores in the bowel movement review questionnaire (lubiprostone 13.3±4.91 to 6.6±1.11, placebo 13.4±4.8 to 10.2±6.5; p<0.05) reported.  2°: AE in intervention vs. control group:  any loose stool: 12 vs. 1  persistent diarrhea: 1 vs. 0  abdominal pain: 2 vs. 5  stool discoloration: 1 vs. 0  indigestion: 0 vs. 1  day time sleepiness: 1 vs. 1  fatigue: 1 vs. 0  fever: 0 vs. 1  rash: 1 vs. 0  bladder incontinence: 0 vs. 1  hallucinations: 0 vs. 2 | 18/22 |
| Parkinson Study Group | Parkinsonism Relat Disord | USA | Randomized, double-blind, placebo-controlled, trial | 37 PD-patients with constipation not controlled by available medication | Rome III criteria | 100mcg of relamorelin subcut once daily | 1°: No significant differences between both groups detected with regard to BMs/week (relamorelin 4.1±1.1 to 4.3±1.5 vs. placebo remaining at 4.0±2.1; CI95%=0.81,1.54; p=0.5), spontaneous BMs/week (relamorelin 4.1±1.1 to 4.3±1.5 vs. placebo 3.8±2.3 to 3.9±2.1; CI95%=0.81,1.53; p=0.52), complete BMs/week (relamorelin 1.3±1.1 to 2.1±1.7 vs. placebo 1.6±1.0 to 1.3±1.2; 95%CI=0.65, 3.01; p=0.39), complete spontaneous BMs/week (relamorelin 1.3±1.1 to 2.1±1.7 vs. placebo 1.6±1.0 to 1.3±1.2; 95%CI=0.65, 3.01; p=0.39), days with no BMs (relamorelin 2.9±1.1 to 2.7±1.5 vs. placebo 3.0±2.1 to 3.0±2.1; p=0.78).  2°: No serious AE reported | 16/22 |
| Sakakibara (2005) | Mov. Disord. | Japan | Before-and-after study | 6 PD-patients with constipation  (4 MSA-patients disregarded) | Not specified | 15 g Dai-Kenchu-To TDS (50% ginger, 30% “Nin-jin” [ginseng], and 20% “Sansho” [Japanese pepper, Zanthoxylum]) | 1° Total CTT was reduced from 88.7 at baseline to 64.6 hours after 12 weeks (NS), right colon 26.9 to 18.1 h (NS), left colon 20.7 to 23.3 h, rectosigmoid 41.1 to 23.3 h (NS). Rectal pressure increased from 12.0 to 14.5cmH2O at rest (NS) and 3.2 to 12.2 5 cmH2O during defecation (NS), no significant differences in anal pressure (37.2 to 38.7 cmH2O) and post-defecation residual (161.7 to 67.0 ml).  2°: Except for bitter taste well-tolerated | 10/16 |
| Sullivan (2006) | Mov. Disord. | USA | Randomized, double-blind, placebo-controlled trial | 15 PD-patients with constipation | Rome II criteria | Tegaserod 6 mg BID | 8 subjects received tegaserod, 7 placebo  1°:  total SGA (baseline, after 1 month): tegaserod 9.1±4.4 to 8.3±4.0, placebo 6.2±3.7 to 8.7±3.9 (p=0.10)  bothersome constipation (baseline, after 1 month): tegaserod 3.3±2.0 to 2.8±1.7, placebo 2.2±1.5 to 3.0±1.5 (p=0.14)  SGA of abdominal pain and discomfort (baseline, after 1 month): tegaserod 2.8±1.5 to 2.5±1.4, placebo 1.7±1.9 to 2.7±1.5 (p=0.30)  SGA of satisfaction (baseline, after 1 month): tegaserod 3.1±1.1 to 3.0±1.1, placebo 2.3±1.0 to 3.0±1.1 (p=0.10)  UPDRS (baseline, after 1 month): tegaserod 42.1±22.5 to 39.8±25.7, placebo 33.8±10.4 to 37.0±11.6 (p=0.32)  2°: no side effects | 13/22 |
| Tateno (2011) | Parkinsonism  Relat Disord | Japan | Before-and-after study | 18 de novo PD-patients with constipation | According to a questionnaire on pelvic organ function (Sakakibara et al., 2001) | 200/20 mg levodopa/  carbidopa BID | 1°: no statically significant subjective improvement in bowel frequency and defecating difficulties. CTT did not change significantly for any colon part: total colon 49.3 to 56.7 hours, right colon 10.0 to 9.7 hours, left colon 12.5 to 15.4 hours, rectosigmoid 26.6 to 31.6 hours. In resting state, no change in rectoanal videomanometry (anal sphincter pressure, anal squeeze pressure, abdominal pressure with coughing or straining). During rectal filling levodopa significantly reduced the first sensation (178.6 ml – 121.3 ml) (p<0.05). An enlargement of the amplitude of spontaneous phasic rectal contraction was not statistically significant. During defecation, levodopa significantly lessened the amplitude in paradoxical sphincter contraction (29.7 cmH2O to -7.1 cmH2O, p<0.01). The amplitude of rectal contraction and of abdominal strain did not change statistically significantly. Overall, levodopa significantly lessened post-defecation residuals (142.2 ml – 53.9 ml) (p<0.05).  2°: no adverse GIT effects | 12/16 |
| Zangaglia (2007) | Mov. Disord. | Italy | Randomized, double-blind, placebo-controlled trial | 57 PD patients with constipation | Rome II criteria | Macrogol (7.3-21.9g daily) | 1°: Clinical evaluations at baseline, after 4 and 8 weeks, treatment efficacy defined as complete relief of the symptom or a marked improvement of two out of four clinical indicators: stool frequency, straining (0-3), stool consistency (0-100), or use of rectal laxatives as a rescue therapy:  responder rates in active control group 78.3% vs. 25.0% at four weeks (p=0.0003) and 80.0% vs. 30.4% at eight weeks (p=0.0012) for macrogol and placebo, respectively.  Frequency of bowel movements increased significantly from 1.9±0.56 to 5.7±2.3 (4 weeks, p=0.0002) and 6.6±2.7 (8 weeks, p=0.0004) for macrogol and from 2.0±0.61 to 3.4±1.7 (4 weeks, p=0.0009) and 3.7±1.9 (8 weeks, p=0.003). Comparison results in a significantly higher number of evacuations in the macrogol than in the placebo group at 4 and 8 weeks (both p=0.002).  No patient presented very hard stools at any time point, 1 patient (6.3%)  presented hard stools (at week 8); 12 patients (75%) reported formed stools at week 8, and 3 (18%) reported  unformed stools. Comparison of treatments revealed significant improvement in stool consistency  at week 4 (P=0.001) and at  week 8 (P=0.000).  Straining reduced at 4 weeks versus baseline with macrogol (18.5±24;p=0.008) and placebo (30,7±29.7; p=0.015), no significant difference at 8 weeks  Stool consistency was improved in with macrogol at 4 and 8 weeks (p=0.002 and p=0.000).  Rescue treatment with rectal laxatives was recorded for 2 (12.5%) patients on placebo but none in the macrogol group.  2°: 2 withdrawals due to nausea and diarrhea in macrogol group | 16/22 |
| Abbreviations: AE=adverse events, BID=bis in die (twice a day), BM=bowel movement, CBM= complete bowel movement, CFU=colony forming units, CI=confidence interval, h=hours, MSA=Multiple System Atrophy, NS=not significant, OR=odds ratio, PD=Parkinson’s disease, TDS=ter die sumendum (three times a day) | | | | | | | | |
